# Medical educators’ perspectives on the barriers and enablers of teaching public health in the undergraduate medical schools: a systematic review

**DOI:** 10.1080/16549716.2022.2106052

**Published:** 2022-09-05

**Authors:** Nurhira Abdul Kadir, Heike Schütze

**Affiliations:** a Graduate School of Medicine, Faculty of Science, Medicine and Health, University of Wollongong, Wollongong, New South Wales, Australia; b Centre for Primary Health Care and Equity, Faculty of Medicine, University of New South Wales Australia, Kensington, New South Wales, Australia

**Keywords:** Medical curricula, medical education, medical educator, public health, undergraduate medical schools

## Abstract

**Background:**

Having relevant public health content in the undergraduate medical curriculum is critical to preparing medical doctors for emerging health issues and increased public health roles. Medical educators are central to this effort.

**Objective:**

This systematic review synthesises the most relevant and up-to-date evidence on medical educators’ perspectives regarding the barriers and enablers on incorporating public health teaching in the undergraduate medical curricula.

**Methods:**

Seven databases were searched for articles published between 1 January 2010 and 31 December 2021. Articles were included if they were available in full-text English or Indonesian language, peer-reviewed, and focused on medical educators’ perspectives on teaching public health in the undergraduate medical curricula. Findings were integrated to answer the review question using thematic analysis.

**Results:**

Twenty-nine articles were included in the final review. Three major themes emerged: (i) space in the medical curricula, (ii) confidence/capabilities of medical educators, and (iii) institutional support. Overcrowded curricula, lack of consensus about the scope and level of public health to incorporate into teaching, ensuring the quality and the relevance of content with what is required in real practice, as well as inadequate institutional support are major challenges in teaching public health to medical students.

**Conclusions:**

Integrating public health into other subjects is largely seen as a solution. This requires strong institutional support in the form of financial, logistic, and technical support; structured training for medical educators on how to incorporate the content into their subjects; and a recognition of the important role that public health educators play.

## Background

The undergraduate level in medical education provides an ideal opportunity to teach public health [1,2]. Doctors appreciate and value public health more if they are exposed to interesting, relevant, and effective public health learning during their undergraduate medical study [2]. The significance of having public health in the undergraduate medical curricula is generally acknowledged [3]. However, incorporating public health into the medical degree is difficult for a variety of reasons, starting from the conceptual phase in curriculum development, through to curriculum delivery, assessments on students’ performances and competence, and the evaluation of the robustness of the content [4].

Public health as a concept is very broad [5] and means different things to different people, ranging from specific health issues of individual populations, governmental health services, the health of the public in general, to issues affecting the public’s health in general such as climate change [6,7]. Therefore, it is often not easy for medical educators to see how or where to include public health into the medical curriculum, which is dominated by clinical and biomedical worldviews [8,9]. Finding the justification to increase the representation of public health in an already overcrowded medical curriculum has been a challenge for some time [8,10], especially because adding content in any one area can mean reducing the content in another, and assessing public health knowledge and competence needs complex approaches that incur a time cost [10,11].

Challenges in conceptualising public health as part of medical curricula can impact opportunities to incorporate, increase, or improve public health [2,8]. This risks the curriculum, not adequately preparing the doctors for the work they will face in the field once graduated, let alone keeping them prepared for emerging public health issues such as new communicable diseases and the changing dynamics within the social determinants of health in local and global contexts [3]. An obvious recent example of this is the COVID-19 global pandemic where medical schools had to include not only a significant amount of clinical and scientific content related to COVID-19 but also develop students’ understanding of the associated societal aspects, such as strain on an underfunded public health system, vaccine hesitancy, mental health issues among children, failures in disaster preparedness, and how the social determinants of health also play a part [3,8,9,12–15].

Developing medical students’ interest and understanding of population-wide health issues instead of just individual patient presentations is challenging [16] and requires medical educators to have sufficient knowledge, skills, and a willingness to teach public health [17]. Medical educators need to be able to prepare medical students to face multiple challenges in public health, including the increasing number of chronic and non-communicable diseases, an aging population, and rapid advances in technology and information transfer [18].

Teaching any topic, including public health subjects, is closely related to medical educators’ beliefs on the impact of teaching [19]. These beliefs influence medical educators’ teaching practices, including what topics they will cover, instructional methods they will use, the extent to which they can support their students achieve the learning objectives, and how they will assess students’ knowledge and skills [20].

Whilst medical educators are key to public health teaching, they are rarely the focus of research related to teaching public health, and studies consolidating the evidence about medical educators’ perspectives on public health teaching are few in number. Most of the literature related to teaching public health focuses on medical students as participants or innovations made in public health education [21,22]. Research that specifically focuses on medical educators’ attitudes and beliefs about teaching public health is critical to improving the quality of public health education [23,24]. This review therefore answers the specific question: “*What are medical educators’ perspectives regarding the barriers and enablers to teaching public health in the undergraduate medical curriculum*?’

## Methods

This review has been registered with PROSPERO (Reg No: CRD42021237971). The Preferred Reporting Items for Systematic Reviews and Meta-Analyses (PRISMA) [25] method was used for this review: identification and extraction, screening, assessing the eligibility of articles, data analysis, and synthesis of findings.

### Literature search

Seven databases were searched: Scopus was searched independently; Academic Search Complete, APA Psychinfo, CINAHL, ERIC, Medline, and SOCIndex were searched using the EBSCOHost platform, which searches multiple databases at the same time. These databases are chosen as they provided the most recent and up-to-date literature on the topic. To ensure the best possible chance of capturing all relevant results, keywords were selected using a modified version of the PICO (Population, Intervention/Interest, Comparator, Outcome) framework [26]. Both authors constructed and agreed on the keywords with the assistance of an experienced university librarian. The wildcard ‘*’ was used to allow for spelling variations. To ensure that all relevant literature was captured, keywords within the same element were combined using the Boolean operator ‘OR’; the search was then focused by combining the results using ‘AND’ (see Table 1 for the search string).

**Table 1.** Search keywords and search string.

PICO element	Search string
Population	(Lecturer OR Professor OR Teacher OR Educator)
	AND
Interest	(‘Public Health’ OR ‘Population Health’ OR ‘Social Determinants of Health’ OR ‘Health Advocacy’)
	AND
Outcome	(Attitudes OR Beliefs OR Perceptions OR Views OR Opinion)
	AND
Other	(Medic* Curricul* OR ‘Medic* Education’)

The following limits were applied: peer-reviewed, available in full text, and published in English or Indonesian between 1 January 2010 and 31 December 2021.

### Eligibility criteria

#### Inclusion criteria

Papers were eligible if they were primary studies (cohort studies, randomised controlled trials, case–control studies, cross-sectional studies, and qualitative studies) that explored medical educators’ perspectives of teaching public health in the undergraduate medical schools. Public health is very broad, and there is a call to have a clear definition and scope defined [27]. This definition is beyond the scope of this study. For the purposes of this review, the authors have adopted the broad definition of public health given by Winslow [28, p. 335–338]: the ‘science and art of preventing disease, prolonging life and promoting physical and mental health and well-being.’

#### Exclusion criteria

Papers were ineligible if they were commentaries, letters, editorials, opinion pieces, and reviews; had a focus on medical students’ or non-educators’ perspectives of teaching public health; had a focus on teaching non-public health subjects; or focused on postgraduate teaching. In the case where participant samples were mixed, for example studies that included both medical students and medical educators, papers were only included if the results could be clearly separated. Grey literature was excluded.

### Study selection

Both authors independently used a stepwise procedure to identify relevant papers. NAK performed the initial search and imported articles into Microsoft Excel. Whilst EBSCOHost automatically removes exact match duplicates, further duplicates not captured by EBSCOHost were removed manually. NAK screened the articles’ titles and abstracts against the inclusion and exclusion criteria; the full text of the remaining articles was retrieved and further screened against the inclusion/exclusion criteria, and results for exclusion were recorded. HS independently checked the results and compared her results with the first author. Any disagreements between results were discussed and resolved by consensus. A third reviewer was available if consensus could not be reached. NAK scanned the reference lists of any included articles to identify any additional articles that were not captured in the initial search which HS reviewed. Consensus was reached through discussion between both authors.

### Study quality appraisal

NAK and HS independently assessed the rigour of the included articles using the Joanna Briggs Institute (JBI) Critical Appraisal Tools [29]. The JBI suite was selected as it contains 13 checklists, one for each study type, a consistent scoring system (include, exclude, and seek more information) across 13 study types, which greatly aids in making assessments across different study types. Studies deemed to be of poor quality were excluded. Any disagreements between results were discussed and resolved by consensus. A third reviewer was available if consensus could not be reached. The appraisal of the quality of the included articles is provided in Supplementary Table S1.

### Data extraction and synthesis of evidence

The following data were extracted into a Microsoft Excel spreadsheet: first author, year, country, study type, aim, sample, methods, and conclusion. The results from the quantitative, qualitative, and mixed methods studies were synthesised using a convergent qualitative synthesis design [30], allowing for a comprehensive picture of the issues relating to the review question [30]. First, the quantitative results were extracted and placed into a table and converted to a meaningful narrative summary to allow for coding [31]. Next, a narrative summary of the qualitative results was extracted into the same table. These were then coded and analysed qualitatively: both authors worked together to create the initial code frame, cross-coded the narrative findings, and organised them into overarching themes based on the review question using Braun and Clarke’s thematic analysis framework [32,33]. Detailed steps for the thematic analysis and examples are provided in Supplementary Table S2.

A number of steps were undertaken to increase trustworthiness according to the criteria outlined by Lincoln and Guba [34,35] as follows. Credibility: the authors read and re-read the articles to become immersed in the data (prolonged engagement) and by having more than one researcher perform the screening and analysis (researcher triangulation and peer debriefing). Transferability: A thick description of codes and themes was provided when developing the codes and themes (see also Supplementary Table S2). Dependability and Confirmability: A reflexive journal was kept when developing codes and themes; researcher triangulation and peer debriefing were also used.

## Results

### Study selection

**Figure 1.**
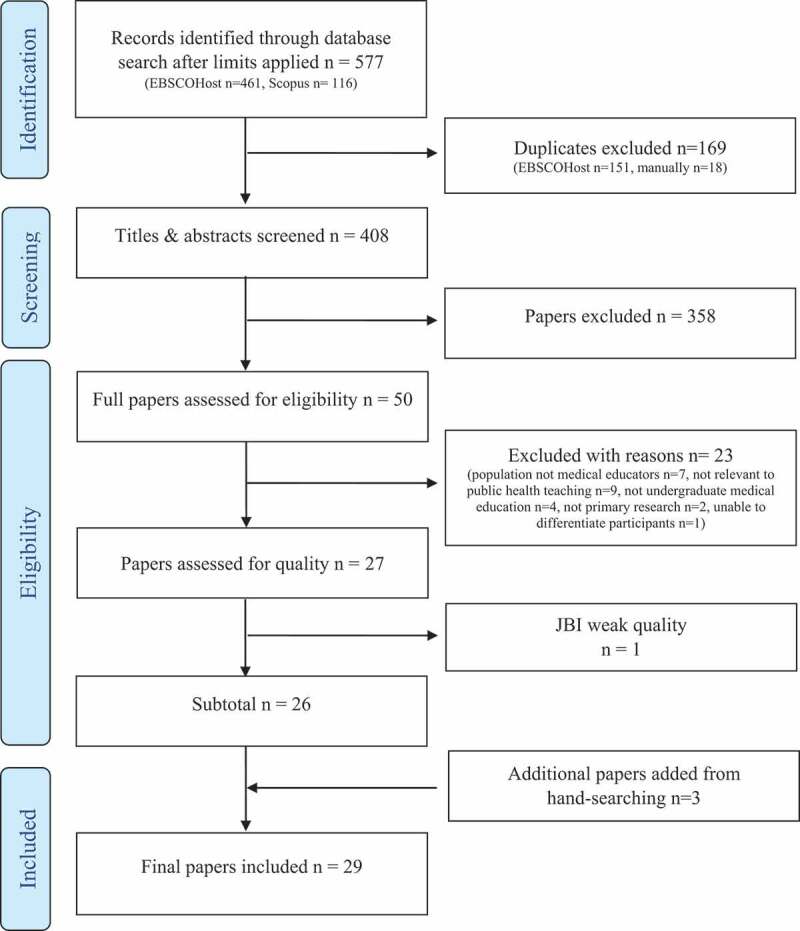
PRISMA flowchart of the selection process.

The initial search yielded 577 results after limits were applied (EBSCOHost n-461, Scopus n = 116). One hundred and fifty-one duplicates were automatically removed in EBSCOHost. The results from the EBSCOHost and Scopus searches were then combined into one Excel file, and a further 18 duplicates were removed manually, leaving 408 papers. All results were in English, and no Indonesian translation was necessary. After reviewing the titles and abstracts against the inclusion criteria, 358 papers were excluded. The full text of the remaining 50 papers was examined and a further 23 were excluded. The remaining 27 papers were assessed for quality using the JBI checklists, resulting in one being excluded due to poor methodological quality. Another three papers were included from hand-searching, bringing the final total of papers included in the review to 29 (see Figure 1).

### Study characteristics

Of the 29 included papers, 21 were conducted in a single country (one each in Indonesia, Brazil, New Zealand, Uganda, Norway, Germany, Bangladesh, South Korea, Malawi, China, and Sweden; two each in the USA, South Africa, and the Netherlands; and three each in Australia and the UK); five studies were conducted in two different countries and three involved multiple countries in Europe and the US (see Table 2). Sixteen of the articles were published in the last 5 years. Fifteen studies had medical educators only as participants; 14 had a mix of medical educators with either students or other experts. There were two quantitative, 22 qualitative and five mixed-methods papers (see Table 2). Eight mentioned how public health is structured in the curriculum (see Table 2).

**Table 2.** Summary of included papers.

First Author & Year, Country	Study aim	Data collection,Sample,Analysis	Results/Conclusion	Curriculum structure,Themes
Abbott 2020,Australia, New Zealand and Canada	To examine ME and student views of clinical learning in correctional settings, with a focus on the Australian context.	Semi-structured interviews.19 MEs, 17 medical students, 2 GP registrars.Thematic Analysis.	Clinical placements provided were beneficial for learning about managing complex consultations, mental health and substance use disorders, and overcoming anxiety related to interacting with people in prison.	a, c1, 2, 3
Ahern 2017,Australia	To explore stakeholders’ perceptions of shared learning in general practices in northern NSW, Australia.	Semi-structured interview.11 MEs, 8 registrars, 2 trainees, 8 students and 4 practice managers.Thematic analysis.	General practice played a critical role in shared learning, including improved morale, collegiality, financial rewards, sharing of resources and experience, and reduced social and professional workload and isolation.	2, 3
Ball 2010,Australia and New Zealand	To explore the perceptions of GP MEs regarding the role of GPs in general practice nutrition care, competencies required, and teaching and learning strategies	Semi-structured interviews.20 MEs Thematic analysis.	Nutrition was an important yet superficially addressed component of health care in general practice. Barriers to nutrition care included a lack of time, financial disincentives, inadequate skills and training, and ambiguous attitudes and perceptions about the role of GPs in nutrition care.	a1, 2, 3
Barber 2019,UK	To understand facilitators and barriers to GP engagement with undergraduate education.	Semi-structured interviews.24 MEs.Thematic Analysis.	GPs that engaged with undergraduate medical education negotiated membership in three communities of practice: (i) clinical practice, (ii) the medical school, and (iii) teaching.	a1, 3
Berkenblit 2012,USA	To explore MEs’ attitudes, barriers, and behaviours regarding advocating routine HIV testing to their trainees.	Cross-sectional self-completed survey.335 MEs.Bivariate and multivariable logistic regression.	MEs who are clinicians have a unique role in disseminating Centres for Disease Control recommendations as they impact the knowledge and attitudes of newly practising physicians. Despite awareness of these recommendations, many clinicians do not recommend universal HIV testing to trainees.	1, 2, 3
Claramita 2019,Indonesia	To develop a Community-Based Education framework for undergraduate medical education to better engage students and MEs in primary health care.	Interviews.18 MEs.Grounded theory.	A new Community-Based Education framework was identified at three levels: micro-, meso-, and macro-curriculum. Different components in the three levels were explored.	a2
Clithero-Eridon 2020,South Africa	To assess the degree to which medical students, preceptors and community mentors understand the concept of social accountability.	Survey.86 MEs, 256 students.Qualitative analytic approach.	Most participants understood social accountability as requiring an action or set of actions and an awareness one must have about the needs of their patients, community or society. However, participants did not identify to whom the accountable party should answer to.	2, 3
da Silva 2018,Brazil	To analyse ME participation and integration in healthcare services in Brazil.	Survey.MEs in 41 undergraduate medical courses.Content analysis.	ME participation and teaching service integration are limited to the accomplishment of actions. Strengthening teaching-service-community integration policies requires investment in teaching professionals and recognition of their social role as agents of change.	1, 2, 3
Duncan 2011,UK	To explore ME views on the nature of ‘good practice’ in health care and how this could be connected to the persona of the ‘good practitioner’.	Focus group discussion.11MEs.Thematic content analysis.	MEs’ views centred on four aspects: (1) difficulties in explaining ‘the good practitioner’, (2) the significance of systems and contexts in understanding this area, (3) the place of consultation and diagnosis in conceptions of ‘good practice’ and ‘the good practitioner’, and (4) the realisation of the ‘practitioner who is good’ into good practices.	1, 2, 3
Friedman 2013,India and Brazil	To explore how MEs understand and apply existing theories about the relationship between education and health.	Survey.139 MEs.Descriptive statistics.	Pathways to improved societal health via increased quality and quantity of education were identified and require faculty development, networks and partnerships with other institutions.	1, 3
Galukande, 2012,Uganda	To explore perceptions of senior MEs and students on the concept and evidence of expression of social accountability.	Semi-structured interviews.10 MEs, 2 medical students.Thematic analysis.	Despite a general unfamiliarity of social accountability, medical schools had been socially accountable. There is a need for increased awareness by articulating a model to guide further implementation.	1, 3
Gran 2016,Norway	To explore GP MEs’ and medical students’ experiences with giving and receiving supervision and feedback during a clerkship in general practice.	Focus group discussions.21 MEs, 9 medical students.Thematic analysis.	Mutual trust builds a learning environment where supervision and feedback may be given. Structured tools may promote feedback, reflection, and learning.	2, 3
Gum 2013,Australia	To describe how a partnership between primary health service and a university-led to developing an interprofessional capabilities framework.	Action research. Five focus group discussions.11 MEs, 14 health service providers).Thematic analysis.	The development of a capability framework for inter-professional practice is needed. The framework can guide curricula to assist the incorporation of interprofessional capabilities into students’ learning and practice.	1, 3
Havemann 2018,Germany	To explore Global Health MEs’ views on: (1) What is, (2) What belongs to, and (3) How can global health be taught?	In-depth interviews.11 MEs.Grounded theory.	Global health is understood as an umbrella term and is multidisciplinary. At its core is the supra-territorial determinants of health.	1, 2
Hordijk 2019,8 EU countries, Ireland, UK	To devise a framework of competencies for diversity teaching.	Delphi technique (three rounds).36 MEs	The final framework consisted of 10 competencies that were seen as essential for all MEs.	1, 2, 3
Khan 2012,Bangladesh	To develop a community-based ophthalmology curriculum for the undergraduate medical course in Bangladesh	Modified Delphi technique.60 MEs, 340 eye specialists.	A community and need-based ophthalmology curriculum for an undergraduate medical course in Bangladesh was developed.	a1, 3
Loh 2013,Brazil and India	To identify educators’ perceptions of elements that influence sustainability in innovative projects and those identified in project sustainability literature.	Self-complete questionnaire.139 MEs.Iterative coding.	Two main factors of innovative project sustainability were identified: 1. Project-level factors (project design, monitoring and evaluation, stakeholder support, and project outcomes) 2. Context level factors (institutional, governmental, peer support, self-motivation).	3
Mudarikwa 2010,Australia	To evaluate a community-based practice program conducted at Gippsland Medical School, Monash University.	Mixed methods. Focus group discussions and interviews.19 MEs, 57 medical students.Thematic analysis.	Community MEs viewed the program as a valuable platform for mutual learning for all parties involved, with students gaining real-life experience. Challenges included formulating and conducting a research project and contextualisation of didactic material at community sites.	1, 2, 3
Ottenhoff-de Jonge 2019,USA, Netherlands	To explore ME beliefs about ME qualities.	Interviews.26 MEs.Deductive and inductive analysis.	Four profiles of MEs were identified: the ‘Inspirer’, ‘Role-model’, ‘Practitioner’, and ‘Critic’.	2, 3
Ottenhoff-de Jonge 2021,USA and Netherlands	To describe MEs’ beliefs about teaching, learning, and knowledge specifically adapted to the medical education context.	In-depth interviews.26 MEs.Deductive analysis.	Multiple adaptations and refinements to the Samuelowicz and Bain beliefs were necessary to align the framework to the context of medical education. The refined belief dimensions and belief orientations enable a comprehensive description of the educational beliefs of MEs.	2
Park 2021,South Korea	To explore how the Korean Medical Colleges responded to the COVID-19 pandemic and how this influenced current and future medical education.	Survey.37 MEs.Modified thematic analysis.	The Deans’ perspective aligned more closely with Generation Z medical students. There is a need to invest in faculty development so medical educators can be competent in diverse information and technology learning platforms.	3
Sawatsky 2016,Malawi	To explore the role of culture in the development and maintenance of mentoring relationships in the University of Malawi College of Medicine.	In-depth & semi-structured interviews.11 MEs, 13 medical students, 12 interns, 10 registrars.Thematic analysis.	Themes identified: 1. intrapersonal (Malawian politeness, mentoring needs, and friendliness and willingness to help); 2. interpersonal (understanding the role of the mentor, respect for elders, personal and professional boundaries, and perceptions of others), and 3. institutional (the supervisor versus mentor, time pressures, tension about the scope of training, and the mentoring cycle).	1, 3
Seeleman 2011,Netherlands	To develop a recommendation to teach communication skills based on the experiences of members of a Dutch NMVO Special Interest Group on’Diversity’	Survey.23 MEs.Thematic analysis.	Training in communication skills for consultation with ethnic minority patients cannot be separated from teaching issues of awareness and knowledge. The shared views on the content of these communication training are in line with general patient-centred approaches.	1, 2, 3
Shi 2019,China	To construct a general model of the competencies required by Chinese public health physicians, lay the foundation for promoting reform of public health education in China, and revise the testing and grading system of public health courses	Mixed-methods.Modified Delphi technique.85 MEs.	Sixty public health competency areas were identified. There were significant differences in the perceived importance of the 60 core competencies between public health professionals and public health education specialists. The model built from the core competencies ended up with seven competency dimensions.	1, 2
Sorensen 2019,USA and 12 EU countries	To provide a snapshot of the role of cultural competence in European medical educational programmes	Survey.MEs in 12 European universities.Thematic analysis.	There are major deficiencies in the commitment and practice within the participating medical schools and clear potential for major improvements regarding cultural competence in programmes.	a1, 2, 3
Von Below 2015,Sweden	To explore GP ME views tutors on the characteristics of a skilled GP tutor.	Focus group discussions.20 MEs.Content analysis.	Three main characteristics of skilled GP tutors were identified: (1) Professional as GP and ambassador to general practice, (2) Committed and student-centred educator, and (3) Coordinator of the learning environment.	2
Walpole 2015,UK	To engage healthcare students, healthcare educators and other key stakeholders to develop learning objectives for undergraduate and postgraduate medical education.	Delphi technique (three rounds).35 MEs, 17 medical students, and 12 non-medical educators.	This is the first attempt to define consensus learning objectives for medical students about environmental sustainability. Allowing a wide range of stakeholders to comment on multiple document iterations stimulated their engagement with the issues raised and ownership of the resulting learning objectives.	a, b1, 2, 3
Waterval 2018,Netherlands	To investigate cross-border medical curriculum partnerships by exploring MEs’ experiences at the recipient institution who have a key role in delivering the program.	Q study survey.24 MEs.Factor analysis.	Three viewpoints emerged: (1) connectedness with the partner institution, trust in the quality of the curriculum, and appreciation of interinstitutional relationships; (2) the partnership’s attractiveness because of the career opportunities it offers; and (3) concerns over the quality of graduates due to content the practical feasibility of s partnerships.	3
Wolvaardt 2013,South Africa	To explore the conceptualisation and implementation of public health in a medical curriculum in South Africa.	Mixed methods. Interviews and focus groups.11 MEs.	Multiple concurrent understandings of public health were identified along with educational tensions, constraints, and points of connection between medicine and public health in the curriculum.	b, d1, 2, 3

Key: GP: general practitioner; ME: Medical educator; a: integrated; b: discrete; c: compulsory; d: elective; 1: Space in the medical curricula; 2: Confidence/capabilities of medical educators; 3: Institutional support

### Thematic analysis

Three major themes emerged: (i) space in the medical curricula, (ii) confidence/capabilities of medical educators, and (iii) institutional support. The themes and their subthemes are discussed below. Table 2 includes a key of the theme of each paper according to the authors’ thematic analysis.

#### Space in the medical curricula

This theme covers aspects involved in making space for public health in medical curricula. Included are three subthemes: issues in the definition and scope of public health; compulsory versus elective subjects; and integrated versus discrete subjects.

Medical educators consistently identified public health as an essential part of patient care and to improve the health of the overall population [10,24,36]. Conventional medical curricula were not seen to adequately prepare doctors to play their role in addressing the social determinants of health, including advocating for people’s cultural, economic, and social needs [8,10,37]. However, the nature of public health was considered different from the mainstream biomedical world, which made it challenging when allocating space to teach it in the dense medical curricula [8,10]. Finding the justification to increase the representation of public health in an already overcrowded medical curriculum has long been a challenge [8,10], especially because adding content in any one area can mean reducing the content in another [10].

##### Issues in the definition and scope of public health

One of the most important challenges in public health teaching lies in the difficulty in clearly defining ‘public health’ [27]. Medical educators have a different understanding of the definition and the scope of public health and its derivates, for example, population health, global health, community health, and international health [27,38]. Public health is indeed a very broad area, and different aspects are more pertinent in different regions, which potentially makes choosing topics to teach complicated [10,24]. As a result, there have been multiple versions of the public health syllabus and different deliveries of it [10].

Misalignment has become a central issue in public health teaching, and medical educators have expressed challenges in aligning their teaching with ways that could be implemented in real practice or that new graduates would find relevant [10,39]. In the general practice setting, medical educators who were also general practitioners (GPs) expressed the impacts of learning without having a clear scope:

As GPs, we get a lot of stuff dumped on us. Everybody thinks that by educating us they are going to certainly solve the world’s problems. I guess from our side of the fence it feels that everybody expects us to know everything about everything. Can you imagine what that must be like? Medical educator, GP researcher, Australia/ New Zealand [10].

To prevent the unnecessary wide variation in topics taught, it is important that, at least at the national level, there is a consensus on what the learning objectives should be, and a degree of flexibility should be given to medical educators to choose what topics or area to include [13,27,40–42]. This is because public health is very contextual. For example, in China, the set of competencies defined in a nation-wide survey did not include policy development, partnership and management skills, and cross-cultural competencies [41]. The authors suggested, ‘This may partly be due to the influence of Chinese traditional culture and the limitation of people’s perceptions of public health workers’ [41]. Meanwhile, in the UK, nationwide surveys to obtain consensus on the learning objectives of public health in the medical curricula helped identify and address the deficit in the existing curricula and therefore enabled medical educators to choose topics [13].

##### Compulsory versus elective subject delivery

Subjects can be either compulsory or elective [8,27]. Making public health compulsory for students to learn was seen as the way to secure space for it in the curriculum [13]. Whilst medical educators agreed that teaching public health is necessary and making it compulsory is generally acceptable, there was limited consensus on what topics should be included and how much [10,43], and initiatives to obtain nationwide consensus are rare [13].

Wolvaardt [8], however, argues that because the medical curriculum is already overcrowded, the solution is to provide public health subjects as electives, thereby allowing them to occupy an ‘uncontested space’ in the medical curricula [8,p.120]. Wolvaardt [8] further states that when public health was offered as elective subjects, students felt more encouraged to learn public health. However, offering public health as elective subjects needs to take into consideration the possibility of low motivation of students who perceive public health as not being relevant to their medical degree [8]. Medical educators can improve student motivation for choosing public health electives by providing sufficient promotion and remaining actively involved, including during fieldwork [8].

##### Discrete versus integrated subject delivery

Another debatable aspect about teaching public health is whether to deliver it as a standalone subject or to integrate it into other subjects [10,13]. Medical educators felt that teaching public health as discrete subjects was important to ensure that students learned the content, especially when there was no way to ensure that it was sufficiently integrated in other subjects [8].

One important advantage to having discrete public health subjects is that it commonly involves learning with communities either within or outside healthcare settings [8,37,43,44]. Learning with and within communities through community placements is increasingly acknowledged as an important way to learn public health [45]. Community placements are viewed as being beneficial in many ways: they expose students to a wide range of social and health issues; deepen students’ understanding of factors which affect community health needs and outcomes; improve students’ cross-cultural communication skills [8,44]; and encourage students to consider taking up practise in rural settings in the future [8,45].

Although having public health as a discrete subject has been the usual method for incorporating public health into medical curricula, it is integrated public health teaching that has been seen as a solution for issues regarding space in the curricula [10,24,44]. This is because it does not require expanding the number of subjects offered in the curricula, but rather it focuses on increasing the visibility of the public health role and its relevance to biomedical and clinical subjects [10,24]. Integrated public health can occur along the teaching continuum, from curriculum development to assessment or evaluation [37,41].

Medical educators who are not public health educators generally believed that it was important to ensure that there was public health in the medical curriculum because when it was not compulsory for them to integrate public health into their subjects, it was not necessarily a priority [36,37,40]. They also considered that developing public health teaching materials and assessments, and evaluating these, were the responsibility of public health academics, and some felt that public health academics should teach said materials [36].

Integrating public health into non-public health subjects often requires combining multiple ways of teaching [44]. This makes it attractive to both educators and students as it moves the practice from the conventional teacher-centred focus to teaching that is problem-based, case-based, or team-based [39,43]. Medical educators teaching non-public health subjects expressed their interest in being involved in teaching some public health aspects [10,46,47], for example, improving patient lifestyles or encouraging the adoption of government public health recommendations [10,46,47], the magnitude and epidemiology of diseases, health education and rehabilitation of diseases, improving peoples’ attitudes towards diseases, the welfare of patients, teamwork and leadership, and community-based organisations and activities for addressing diseases [13,24,47]. Some felt that public health should be inserted into every clinical and biomedical subject [27].

It is critical in the development of an integrated public health curriculum that there is input from multiple stakeholders, including students, peer-tutors, doctors, governments, and teaching institutions [13,37,40,41,48]. Given that integration will lead to the inclusion of public health aspects in different subjects, it is important to ensure that the topics taught are not repetitive across subjects, that they are delivered consistently, and that required competencies are adequately defined and mapped to best practice [13,41].

#### Confidence and/or capabilities of the medical educator

Medical educators’ confidence and capacity to teach public health is a significant aspect in public health teaching. Medical educators have to carefully choose the best instructional methods to achieve the pre-determined learning objectives [8]. Teaching public health needs to be approached with non-conventional methods that are able to target the most learning objectives in the shortest time and using the least resources [44]. Included in these is choosing places where teaching can be delivered effectively [8,45]. Currently, public health teaching takes place in classroom settings, in institutions such as hospitals and clinics, and in community settings. However, a study on medical educators teaching in primary care settings showed that they believed that the skills and knowledge offered in primary care were different, equally valid, and, in some ways, more positive and richer than those offered in a hospital environment [48]. Regardless, all settings present different challenges unique to their context. For example, those teaching in general practice settings found that, in relation to their work as a medical educator, they had to negotiate and balance their clinical practice roles, their teaching roles, and their role in medical school communities [48,49]:

To avoid frustration [from too heavy a workload] and to limit other activities … we planned our schedule in advance … and cancelled telephone consultations for the physicians. Medical educator, GP, Sweden [49]

Teaching students off campus requires consideration of several factors, including the time taken to transport students to the field and potential delays in accessing information via internet connections when connectivity is not available [8]. Despite being time- and resource-intensive, exposure to the community was seen as being among the most effective ways to cover different aspects of public health in one setting [45,47].

Variation in the topics taught and coverage of their scope in teaching depend on the capabilities of the medical educators to teach public health [13,27,40–42]. Public health teaching needs to be approached with creativity and intuition, and educators need to be able to teach in different scenarios [27,40,50]. Medical educators need to be able to adapt their teaching to suit the dynamics within the class due to the different abilities of students, different resources available, and different needs in doctor practice [37,40].

Although assessments are set to measure what medical students have learnt about public health, they also provide an important opportunity to enhance learning in the dense medical curricula as they show how well future doctors are able to grasp public health concepts and to implement them in their care [8]. Medical educators need to learn from each other about how to best assess medical students learning achievement [8]. Literature shows that complaints have been made about the disconnection between what is taught in class, how this is assessed, and the reality of practice [10]. This raises questions as to whether or not medical educators fully understand the scope of public health that is critical for doctors to be able to contribute effectively to the health of the population [8,10].

Another important aspect highlighted was the need for students to see real-life examples on the best way to implement public health into practice via role models [19,23,36,46,51–53]. A range of competencies has been identified for medical educators to be good role models in public health: being able to critically reflect on one’s own values, beliefs, ethnicity, cultural backgrounds, and intersectionality; being competent with students when demonstrating how to show empathy and communicate in non-discriminatory and non-stereotyping ways; demonstrating a high knowledge of ethics and the social determinants of health; and assisting students to reflect on patients’ social determinants of health during a clinical encounter [36,51,52]. Primary care medical educators in one study felt that they are appropriate role models as they were able to demonstrate culturally appropriate communication with patients to students in their practice [36]. Demonstrating a high level of knowledge on content has also been shown to be a motivator for students [23].

Medical educators acknowledged that students may have different learning needs and different views and interests about topics taught at medical school [27,51], which required teaching to be personally tailored to individual needs [54]. The way medical educators approach teaching public health is influenced partly by how they view the significance of students in the teaching and learning process and the significance of their own role for students’ learning [19,23,51].

The importance of students’ prior knowledge has been recognised by medical educators. Students’ experiences are valuable, particularly on the teaching and learning process, and on collaborations between educators and students in order to build knowledge constructs [51,54]:

[students and trainees] have often had other careers, [and] bring a whole lot of expertise from other areas and everyone just has different interpersonal skills or interests so you just get different viewpoints on things, so I think it’s more educational basically Medical educator, Australia [54]

What I know about adult learning is that they do best when they are focused on what is important to them, and so if they have identified their own specific learning objectives, and we as the facilitator teacher help them with that, then that is reinforcing and motivating. Medical educator, the Netherlands/ the USA [23]

Although the students’ role in teaching and learning was acknowledged as important, medical educators did not express that students needed to be in control of teaching the content [23]

#### Institutional support

This theme captures several subthemes including medical educator staffing levels, medical educator training, medical educator recognition, faculty infrastructure, faculty policies, and partnerships.

Staffing

At the institutional level, when there are competing needs to allocate resources to teaching, a low priority is placed on teaching public health [36,40]. This is particularly seen in terms of institutional support regarding both the quantity and quality of medical educators [48]. The number of medical educators recruited per year is limited, and public health medical educators are either not on the priority list or not supported enough to choose a teaching career over another career [48]. Although many GPs could potentially be recruited to teach in the primary care setting, efforts made to encourage GPs to choose teaching careers are sometimes inadequate:

Younger GPs are definitely not going into partnership, not going into salaried positions, so there are more and more portfolio GPs, so we have a whole host of GPs here, who are, maybe, quite keen to teach but they don’t have the facilities to do it. Medical educator, GP, the UK [48].

In some cases, although medical schools were capable of recruiting and financing public health projects to strengthen public health teaching and learning, the lack of public health medical educators to support them limited the ability of medical schools to run the projects [55]. Once recruited, attrition of trained staff was another issue that occurred for reasons such as a lack of support offered in terms of training, financial reward/promotion schemes, and recognition [40,50]. These are discussed in more detail further below.

Medical educators often have insufficient time devoted to public health teaching because they have multiple roles including as being a clinician, teacher, researcher, administrator, and mentor [10,37,38,46]:

Too often I have the feeling that if I want to spend time and attention to teaching it has to be done in my own spare time, outside working. Medical educator, the Netherlands/ the UK [19]

This shows how public health teaching has been viewed and remains to be viewed as a marginal or additional task, especially when clinical workloads become an insurmountable barrier [38,48,51]. In addition, those who teach off-campus may feel isolated and disconnected from the school [8,45]. Institutions need to ensure that medical educators are supported enough by recognising that teaching is a central, normalised part of clinical work [48], and that medical educators gain benefits from social interactions across a network of medical educators [8,48].

##### Medical educator training

Institutional support that enhances medical educators’ knowledge needs to be done on a regular basis and address aspects specific to public health [40,46]. Some medical educators complained that there were very few initiatives seen in medical schools to provide training specific to public health teaching, and any training that was undertaken was identified and paid for by the medical educators themselves [40]. Medical educator training facilitated by institutions tended to focus on acquiring a basic qualification in teaching [36,40]. However, for public health teaching, training would need to cover various other aspects such as improving educator knowledge of current government public health policies and initiatives, as well as the potential effectiveness and relevance of teaching public health [10,46]. Non-public health medical educators were more willing to teach public health when they were well informed and felt competent with the material [46,47]. For example, in a study by Berkenblit, Sosman [46], those non-public health medical educators who had sufficient knowledge about the government recommendation about HIV testing, and who believed that it was beneficial for students to get tested, were more willing to become involved in activities that encouraged their students to get tested. Those who refused to promote HIV testing, either had lower knowledge levels about the government program and/or perceived getting tested was insignificant because of low HIV prevalence in the community [46].

Systematic, compulsory, and structured training for integrating public health is important to prepare medical educators to teach in different scenarios [40]. To achieve this, institutions need to have regular and comprehensive evaluations on whether the training helps to improve teaching and learning [8,48].

##### Partnership

Institutional support is needed to create and maintain strong partnerships between medical schools, health-care organisations, and communities [39,53,55]. Often, medical educators have extensive professional networks and are leaders in their field [37]. Institutions should harness these networks and secure financial support to help establish partnerships between medical schools, other external institutions, and communities [39]. These partnerships would provide medical educators an avenue to integrate public health into their subjects as students are exposed to learning with or within communities and external institutions [37,39,44].

##### Recognition

Rewards to medical educators teaching public health may not always be in financial form but can also be in the form of recognition. Universities have the power to advocate that teaching professionals be recognised as an important part of the health system and for their critical role as agents of change [37].

Medical educators also highlighted a lack of recognition in terms of being nominated for institutional awards/prizes, not being given timely feedback or feedback at all from their institutions, or not being recognised by the institution as important members of the faculty and not being invited to events in the medical schools [48].

##### Infrastructure

Infrastructure-related issues are important barriers in teaching public health. Teaching public health off-campus can have issues that may not be faced on-campus. The availability of physical space to accommodate a number of students and support medical educators are a common barrier to teaching within the community setting [48]. Community fatigue can also become an issue when a community repeatedly becomes a teaching site [44]. Financial, logistical, travel, and accommodation issues were also often cited as other challenges in teaching within communities located far away from campus [45]. Students and medical educators did not prefer rural postings for reasons such as separation from their social networks and commitments, and when there was poor communication infrastructure in the area, fear of isolation was also reported [8]. When teaching involves rural placement, medical educators expressed that they needed support by having training or facilities, having pre-departure training, supervision, and feedback, and through smooth communication between the field and campus [44,45,48].

Information and technology such as good internet connection and appropriate screens for displaying teaching materials are in high demand in on-campus settings [56]. Those teaching public health or integrating it into other subjects reported similar issues to those teaching other subjects including the capability of audio-visual equipment to accommodate different teaching methods, fast internet connection for both students and medical educators, and subscription to applications [55,56].

##### Policies

Institutional support may be seen directly or indirectly through their policies, visions, and mission statements. For example, reviving the concept of the social accountability in medical schools may help to improve public health teaching as it reintroduces community, population, society, and the public into medical education discourse, and subsequently into the curriculum [43,53]. It is highly likely that when medical schools start paying more attention to improving social accountability formally by writing this into the medical school vision and mission statements, public health teaching will also benefit [43,53]:

… medical issues are influenced by social issues so they should be integrated in learning and teaching activities, as a leading institution we should strengthen our position in the direction of social accountability … Medical educator, Uganda [43]

Formalising a university policy statement for university support towards public health education is essential [13,47,53] and seen as a strong basis for various activities related to public health teaching ranging from the medical educator recruitment process and resources distribution across subjects to the integration of public health into teaching practice [24,53–55]. Medical educators were more willing to allocate space for public health in their subjects when institution policy dictated that incorporating public health into the curriculum was compulsory [36,37]. The policy can be used to accelerate the integration of public health in teaching plans and practice, learning objectives, assessments, placements, practical sessions, as well as monitoring and evaluation of student activities and learning programmes [44,53].

Institutional support to encourage medical educators to teach and/or integrate public health may not be sufficient if the policy is not comprehensive [47]. Policy non-compliance may occur when policies are considered ill-informed because of insufficient contribution by medical educators [40,46,57]. When it is not possible to involve medical educators in the writing of the policy, the institution should provide close assistance to ensure that the policy is properly implemented [55,57]. Policy implementation requires training, strong leadership, role modelling, and audit and feedback [46,53].

Institutional support is also needed to ensure that public health competencies in subjects match the expectations in the practice setting [10,45] and that medical educators are supported to implement public health within clinical practice [13,40,42,57]. Cross-institutional coordination to develop an undisrupted continuum from teaching and learning to practice within the current health system may help reduce the perception that general practitioners learned too much, but their public health roles were superficial and had little to no impact in improving patient conditions [10].

## Discussion

Three major themes were found regarding medical educators’ views on the barriers and enablers of teaching public health to undergraduate medical students: (i) space in the medical curricula, (ii) confidence/capabilities of medical educators, and (iii) institutional support. These are discussed further below in context to other evidence. Whilst some of the barriers and enablers may be common to other areas in the curriculum [58], public health experiences the barriers worse than other subjects in medical curricula as it is not a clinically based subject area [3].

Overcrowded curricula have long been an issue for teaching in medical schools [15], and this review has highlighted it as a major barrier for medical educators to teach public health. Added to this challenge is the requirement to maintain currency in the curricula. For example, the curricula have had to include a significant amount of clinical and scientific content related to COVID-19, as well as societal aspects such as underfunding of the public health system, vaccine hesitancy, failures in disaster preparedness, and an understanding of the social determinants of health [15]. Unfortunately, when developing medical curricula, public health is often a subject that is sacrificed when trying to negotiate the remaining narrow space in an extraordinarily dense educational environment [3]. While integrating public health into other subjects is often the primary solution offered, this review emphasises the importance of having public health offered as discrete subjects to ensure that public health is actually learned [13]. Public health should be among the core subjects taught; however, if it is well-promoted and implemented, then offering it as an elective is an alternative [8,13].

Having consensus on relevant topics to include can also assist educators to find a way to make space in curricula. Medical schools are often criticised for being unable to discern what is relevant and what is not relevant in their curricula [15]. There needs to be a national consensus on learning objectives that are relevant [13,15,41,59].

Clinical medical educators are clinicians first, and many lack training in teaching skills, let alone training in public health [60]. Lack of guidance on undertaking public health teaching may occur even in well-established universities [52]. For this, substantial institutional support is key. Institutions need to provide proper training, infrastructure, and policy, as well as recognise that teaching is an additional workload for clinicians on top of their clinical load [44,48]. Public health teaching often involves teaching off-campus, and this needs extra attention to ensure that everyone involved is properly supported [51,61]. It is critical that adequate institutional support reaches off-campus institutions to ensure the quality of education is maintained equally both on-campus and off-campus [39,51,62]. Communication between those in the periphery and those on-campus should be done seamlessly [8,51].

Unfortunately, to date, deficits in medical education can sometimes produce doctors who may play a role in exacerbating the inequality in health outcomes in society [62,63]. Significant changes needed to address this are centred on the ability and willingness of medical educators to take relevant steps to improve the quality and focus of medical education [64]. A whole of institution approach is required to provide supportive environments to facilitate interdisciplinary cooperation so that faculties and medical educators can work cooperatively together to make change [58]. Medical educators should be provided training and time to equip them with the necessary capacity to establish ongoing teaching evaluation and improvement cycles [58].

### Strengths and limitations

This review has identified some important views regarding public health teaching. This review was undertaken using rigorous systematic methodology: the PICO framework [26] was used to ensure the best selection of keywords and a consistent search; the gold standard PRISMA method [25] was used to reduce bias during the database search and paper screening; validated critical appraisal tools [29] ensured only high-quality papers were included; and researcher triangulation was used to reduce researcher bias during the screening and analysis. However, there are some limitations. Only published peer-reviewed literature were included, and publication bias could therefore be present. Low quality papers could have contained information relevant to the review question [65]; however, in order to prevent ‘rubbish in – rubbish out’ in the analysis, only high-quality papers were included. Included papers were mostly qualitative due to the qualitative nature of the research question. More quantitative studies might have yielded broader results; however, quantitative studies on teaching public health mostly involved students as participants, not educators. Papers were restricted to those published in English or Indonesian, and it is possible that papers in other languages may have provided additional results. Public health teaching is highly contextual, and its implementation across the world varies for a variety of reasons including differences between medical educators’ perspectives based on their country’s income level and teaching methods, which are outside the scope of this review. As such, some papers may not have been captured in the inclusion/exclusion criteria, and this review may not reflect all practice.

## Conclusion

Despite recognition about the importance of public health in medical curricula, incorporating it remains problematic. Debates on what aspects to teach, how to teach them, and who should do the teaching are ongoing. Involvement of both the government and medical schools is critical to assist the discussion and to develop and implement policy. Strong evidence demonstrating what works and does not work well in public health teaching is the first step to graduating doctors who are more aware of population needs and able to deliver appropriate care in that space.

## Supplementary Material

Supplemental Material

Supplemental Material

## Paper context

Medical educators are key to public health teaching, but studies about their perspectives on teaching public health particularly in the undergraduate medical education setting are few. This review consolidates their views and identifies aspects including overcrowded curricula and lack of consensus about the scope of public health to incorporate into teaching as major challenges. Strong institutional support such as financial, logistic, and training are among ways to improve public health teaching in undergraduate medical schools.
