# Supplementary material for: Medical educators’ perspectives on the barriers and enablers of teaching public health in the undergraduate medical schools: a systematic review
Source: Glob Health Action. 2022 Sep 5;15(1):2106052. doi: 10.1080/16549716.2022.2106052 (PMC9467537; doi:10.1080/16549716.2022.2106052)
Supplement: Supplemental Material [file ZGHA_A_2106052_SM0381.docx]

**Supplementary Table 2. Steps undertaken to synthesize data**

| **Step outlined by Braun and Clarke [32]** | **Process undertaken** |
| --- | --- |
| 1. **Data familiarization**   Read the results line-by-line. Generate “casual observational notes” [32] (p.26). | Read and re-read the results and discussion sections of all included papers line-by-line and sentences relevant to the systematic review research question were highlighted. The quantitative results were extracted into a new table and converted to a meaningful narrative summary to allow for coding. Where the narrative description of the quantitative results was available in the text, it was extracted. For example. Table 4 in Berkenblit [46] was described in that paper as: “*The most common response selected was perception of low local prevalence, followed by more important teaching issues and clinic environment being too busy…”* [46] (p 841-2). |
| 1. **Generating initial codes**   Identify specific segments of datasets relevant to research questions and attach meaningful labels to them. Labels can be a phrase or few words. | Examples of initial coding.  Data extract: *“I personally think it’s better to integrate it [nutrition education] because of this challenge of having such as broad spectrum of nutritional issues and nutritionally related clinical problems that we deal with. How can you possibly or should you separate that? I think most GPs will find that kind of learning more relevant*.” (Medical educator, GP researcher, Australia/New Zealand) [10].  Codes: integrated versus discrete; public health is broad.  Data extract: *“Too often I have the feeling that if I want to spend time and attention to teaching it has to be done in my own spare time, outside working.”* (Medical educator, the Netherlands/ the UK) [19, p. 6].  Codes: limited time for public health teaching; medical educators have multiple roles.  Data extract: “*Those who practiced in clinics with a higher percentage of minority patients and reported a greater estimated local HIV prevalence were more likely to encourage their students to get HIV test (Table 1)*” [46].  Codes: experience influences teaching; role modelling  Data extract: “*While we offer to teachers the possibility to acquire a basic qualification in teaching (by training or by assembling a portfolio), cultural competence training or attention to cultural competence is not a part of this.”* (Medical educator, a European country) [40, p. 4-5]  Codes: availability of training; institutional support; topics for training |
| 1. **Searching for themes**   Examine codes (and their associated data), combine, cluster, or collapse codes together to form the initial themes. Structure all data relevant to each of the potential themes. | Codes were clustered under initial themes. For example:  ‘integrated versus discrete’, ‘compulsory versus elective’, and ‘block mode’ were clustered under the theme ‘teaching practice’;  ‘views on training’, ‘availability of training’, ‘topics for training’, and ‘who pays the training?’ were grouped under ‘medical educator training’;  ‘public health is broad’; ‘overcrowded curricula’; and ‘significance of public health’ were clustered under ’room in the medical curricula’. |
| 1. **Reviewing themes**   Check if the initial themes work in relation to the coded phrases and the entire data set. Check similarities and relationships between themes and identify the sub-themes, which are the characteristics and properties of the identified themes. Generating a thematic ‘map’ of the analysis. | Using the first example above, when reviewing the themes, it was determined that ‘teaching practice’ was not a suitable theme, and ‘room in the medical curricula’, was made as a broader theme in which ‘integrated versus discrete’ and ‘compulsory versus elective’ were subthemes.  Using the last example above, ‘room in the medical curricula’ was large and had several aspects, so was split into subthemes, with ‘public health is broad’ and ‘significance of public health’ combined under the subtheme ‘issues in the definition and scope of public health’. |
| 1. **Defining and naming themes**   Refine the specifics of each theme, and the overall story that the analysis tells, generating clear definitions and names for each theme.  Made linkages among themes and sub- themes. Existing literature was referred to when determining relationships between themes. Distinctive methodological stages (themes) and steps (sub-themes) were defined and named at the end of this stage. | Final themes and subthemes were as follows:   1. Space in the medical curricula    - Issues in the definition and scope of public health    - Compulsory versus elective subject delivery    - Discrete versus integrated subject delivery 2. Confidence/capabilities of medical educators, and 3. institutional support.    - Staffing    - Medical educator training    - Partnership    - Recognition    - Infrastructure    - Policies |
| 1. **Producing the report.**   Select relevant extract examples, final analysis of the selected extracts, relating back of the analysis to the research question and literature. | Producing a scholarly report of the analysis about barriers and enablers of teaching public health. The flow of the report was based on the identified themes and sub-themes. |
