# Supplementary material for: Medical educators’ perspectives on the barriers and enablers of teaching public health in the undergraduate medical schools: a systematic review
Source: Glob Health Action. 2022 Sep 5;15(1):2106052. doi: 10.1080/16549716.2022.2106052 (PMC9467537; doi:10.1080/16549716.2022.2106052)
Supplement: Supplemental Material [file ZGHA_A_2106052_SM0379.docx]

**Supplementary 1. Quality assessment using the JBI quality appraisal tools**

| **Quality appraisal of the quantitative and qualitative components of included papers using JBI quality appraisal tools** | | **Papers** | | | | | | | | | | | | | | | | | | | | | | | | | | | | | |
| --- | --- | --- | --- | --- | --- | --- | --- | --- | --- | --- | --- | --- | --- | --- | --- | --- | --- | --- | --- | --- | --- | --- | --- | --- | --- | --- | --- | --- | --- | --- | --- |
|  |  | Abbott [44] | Ahern [54] | Ball [Ball] | Barber [48] | Berkenblit [46] | Claramita [52] | Clithero-Eridon [53] | da Silva [37] | Diab | Duncan [42] | Friedman [24] | Galukande [43] | Gran [51] | Gum [39] | Havemann [27] | Hordijk [36] | Khan [47] | Loh [55] | Mudarikwa [45] | Ottenhoff-de Jonge [19] | Ottenhoff-de Jonge [23] | Park [56] | Sawatsky [38] | Seeleman [50] | Shi [41] | Sorensen [40] | Von Below [49] | Walpole [13] | Waterval [57] | Wolvaardt [8] |
| Qualitative appraisal | Q1 | Y | Y | Y | Y | - | Y | Y | Y | NC | Y | - | Y | Y | Y | Y | Y | Y | Y | Y | Y | Y | NC | Y | NC | NC | Y | Y | Y | NC | Y |
|  | Q2 | Y | Y | Y | Y | - | Y | Y | Y | NC | Y | - | Y | Y | Y | Y | Y | Y | Y | Y | Y | Y | Y | Y | Y | Y | Y | Y | Y | NC | Y |
|  | Q3 | Y | Y | Y | Y | - | Y | Y | Y | Y | Y | - | Y | Y | Y | Y | Y | Y | Y | Y | Y | Y | Y | Y | Y | Y | Y | Y | Y | Y | Y |
|  | Q4 | Y | Y | Y | Y | - | Y | Y | Y | N | Y | - | Y | Y | Y | Y | Y | NC | Y | Y | Y | Y | Y | Y | Y | Y | Y | Y | Y | Y | Y |
|  | Q5 | Y | Y | Y | Y | - | Y | Y | Y | Y | Y | - | Y | Y | Y | Y | Y | NC | Y | Y | Y | Y | Y | Y | NC | NC | Y | Y | Y | Y | Y |
|  | Q6 | N | N | N | N | - | N | N | N | N | N | - | N | Y | N | NC | N | N | N | N | N | N | N | N | N | N | N | N | NC | NC | Y |
|  | Q7 | NC | N | NC | Y | - | Y | NC | NC | NC | NC | - | NC | Y | Y | NC | NC | N | N | N | NC | Y | NC | Y | Y | NC | N | N | Y | Y | Y |
|  | Q8 | Y | Y | Y | Y | - | Y | Y | Y | N | Y | - | Y | Y | Y | Y | Y | N | Y | Y | Y | Y | Y | Y | N | N | Y | Y | Y | Y | Y |
|  | Q9 | Y | Y | Y | Y | - | NC | N | N | N | Y | - | Y | Y | Y | Y | Y | Y | Y | Y | Y | Y | Y | Y | Y | Y | Y | Y | Y | Y | Y |
|  | Q10 | Y | Y | Y | Y | - | Y | Y | Y | Y | Y | - | Y | Y | Y | Y | Y | Y | Y | Y | Y | Y | Y | Y | Y | Y | Y | Y | Y | Y | Y |
| Cross-Sectional Study appraisal | Q1 | - | - | - | - | Y | - | - | - | - | - | Y | - | - | - | - | Y | Y | - | Y | - | - | - | - | - | # | - | - | Y | - | - |
|  | Q2 | - | - | - | - | Y | - | - | - | - | - | Y | - | - | - | - | Y | Y | - | Y | - | - | - | - | - | # | - | - | Y | - | - |
|  | Q3 | - | - | - | - | Y | - | - | - | - | - | NC | - | - | - | - | Y | NC | - | NC | - | - | - | - | - | # | - | - | NC | - | - |
|  | Q4 | - | - | - | - | Y | - | - | - | - | - | Y | - | - | - | - | NC | NC | - | Y | - | - | - | - | - | # | - | - | Y | - | - |
|  | Q5 | - | - | - | - | NC | - | - | - | - | - | NC | - | - | - | - | NC | NC | - | NC | - | - | - | - | - | # | - | - | NC | - | - |
|  | Q6 | - | - | - | - | NC | - | - | - | - | - | N | - | - | - | - | NC | NC | - | NC | - | - | - | - | - | # | - | - | NC | - | - |
|  | Q7 | - | - | - | - | Y | - | - | - | - | - | Y | - | - | - | - | Y | Y | - | Y | - | - | - | - | - | # | - | - | Y | - | - |
|  | Q8 | - | - | - | - | Y | - | - | - | - | - | NC | - | - | - | - | Y | Y | - | Y | - | - | - | - | - | # | - | - | Y | - | - |
| Include in review based on quality appraisal | | ✓ | ✓ | ✓ | ✓ | ✓ | ✓ | ✓ | ✓ | X | ✓ | ✓ | ✓ | ✓ | ✓ | ✓ | ✓ | ✓ | ✓ | ✓ | ✓ | ✓ | ✓ | ✓ | ✓ | ✓ | ✓ | ✓ | ✓ | ✓ | ✓ |
| Reason (s) | |  |  |  |  |  |  |  |  | Low JBI score |  |  |  |  |  |  |  |  |  |  |  |  |  |  |  |  |  |  |  |  |  |

Key – JBI: Joanna Briggs Institute; ✓: Included; X: Not included; Y: Yes; N: No; NC: Not clear; NA: Not applicable; # : Quantitative results not included in review because participants were not medical educators
